# To feel is to heal—introduction to Emotional Awareness and Expression Therapy

**DOI:** 10.1007/s00482-025-00878-6

**Published:** 2025-03-21

**Authors:** Daniel Maroti, Stephan Frisch, Mark A. Lumley

**Affiliations:** 1 Department of Psychology, Stockholm University, Greta Arwidssons Väg 30, 114 19 Stockholm, Sweden; 2 Klinik für Psychosomatische Medizin und Psychotherapie, Universitätsklinikum Ulm, Ulm, Germany; 3 Sektion Medizinische Psychologie, Klinik für Psychosomatische Medizin und Psychotherapie, Universitätsklinikum Ulm, Ulm, Germany; 4 Department of Psychology, Wayne State University, Detroit, MI USA

**Keywords:** Emotional awareness and expression therapy, Primary pain, Functional somatic syndromes, Functional somatic disorder, Narrative review, Short-term psychodynamic therapy, Persistent physical symptoms, Chronic pain, „Emotional awareness and expression therapy“, Primärer Schmerz, Funktionelle somatische Syndrome, Funktionelle somatische Störung, Narrative Übersichtsarbeit, Psychodynamische Kurzzeittherapie, Anhaltende Körperbeschwerden, Chronischer Schmerz

## Abstract

**Background:**

Persistent physical symptoms (PPS), including (primary) pain, can, according to Emotional Awareness and Expression Therapy (EAET), be precipitated, perpetuated, and prolonged by emotional processes related to unresolved trauma and psychosocial conflicts. EAET is a novel, short-term, psychodynamic- and emotion-focused psychological treatment that targets these etiological factors, intending to substantially reduce or eliminate pain and/or somatic symptoms.

**Objective:**

This article provides an overview of EAET’s theoretical background, core treatment principles, and empirical evidence from randomized controlled trials (RCTs) in alleviating somatic symptoms in people with PPS. Moreover, the potential of EAET and future research directions are discussed.

**Methods:**

We report a selective literature review synthesizing the foundations and treatment characteristics of EAET and the findings from RCTs investigating EAET since 2017.

**Results:**

Grounded in psychodynamic theory, with influences from affective neuroscience and emotion-focused therapy, the core treatment principles are reframing symptom explanations, fostering emotional processing, and facilitating corrective interpersonal experiences. EAET has been implemented in various formats, including individual therapy, group therapy, and internet-administered self-help. Since 2017, seven RCTs have been published, demonstrating efficacy in reducing symptoms, which appears superior to cognitive–behavioral therapy.

**Conclusion:**

EAET is particularly effective for treating chronic (primary) pain conditions such as fibromyalgia and musculoskeletal pain. However, further studies are required to evaluate its long-term efficacy, determine patient characteristics associated with positive outcomes, and better understand its most active mechanisms.

## Abstract (de)

**Hintergrund:**

Anhaltende Körperbeschwerden, einschließlich (primärer) Schmerzen, können – gemäß der „emotional awareness and expression therapy“ (EAET) – durch emotionale Prozesse bei unbearbeiteten Traumata und psychosozialen Konflikten ausgelöst und aufrechterhalten werden. Die EAET ist eine neuartige psychodynamische und emotionsorientierte Kurzzeitpsychotherapie, die auf diese ätiologischen Faktoren fokussiert, um Schmerz und/oder die somatische Symptomatik erheblich zu reduzieren oder zu beseitigen.

**Ziel:**

Der vorliegende Beitrag bietet einen Überblick über den theoretischen Hintergrund der EAET, die zentralen Therapieprinzipien und die empirische Evidenz aus randomisierten, kontrollierten Studien (RCT) zur Linderung somatischer Symptome bei Patienten mit anhaltenden Körperbeschwerden. Darüber hinaus werden das Potenzial der EAET und zukünftige Forschungsfragen diskutiert.

**Methode:**

Wir präsentieren eine selektive Literaturübersicht, welche die Grundlagen und Therapiemerkmale der EAET sowie die Ergebnisse von RCT zur EAET seit 2017 zusammenfasst.

**Ergebnisse:**

Auf Grundlage der psychodynamischen Theorie und mit Einflüssen aus der affektiven Neurowissenschaft und der emotionsorientierten Therapie sind die zentralen Behandlungsprinzipien das Reframing des Krankheitsmodells, die Verbesserung der Emotionsverarbeitung und die Förderung korrigierender interpersoneller Erfahrungen. Die EAET wurde in verschiedenen Formaten umgesetzt (Einzeltherapie, Gruppentherapie und internetbasierte Selbsthilfe). Seit 2017 wurden 7 RCT veröffentlicht, die eine Wirksamkeit hinsichtlich der Symptomreduktion zeigen, die der kognitiven Verhaltenstherapie überlegen zu sein scheint.

**Schlussfolgerung:**

Die EAET ist besonders wirksam bei der Behandlung chronischer (primärer) Schmerzstörungen wie Fibromyalgie und Schmerzen des Bewegungsapparats. Es sind jedoch weitere Studien erforderlich, um die Langzeiteffekte zu bewerten, Patientensubgruppen mit besonderem Therapieansprechen zu identifizieren und die wichtigsten Wirkmechanismen besser zu verstehen.

## Introduction

Patients with persistent physical symptoms (PPS) often report a range of issues, from isolated somatic complaints like chronic back pain to clusters of symptoms such as those seen in fibromyalgia or a broader spectrum of diverse somatic manifestations [1]. PPS are associated with increased healthcare utilization, higher rates of unemployment and sick leave, reduced quality of life, and significant psychiatric comorbidities [2]. Although PPS can have various etiologies, our focus here is on patients with PPS, including those with primary chronic pain, where somatic factors (such as tissue damage or peripheral inflammation), are not believed to be the main cause. Instead, the central nervous system, particularly the predictive processing brain, is the main driver of somatic symptoms.

Emotional Awareness and Expression Therapy (EAET) is a recently developed, emotion-focused, psychodynamic-informed therapy that seeks to address unresolved stressors, conflict, and traumas that are believed to drive or exacerbate PPS [3, 4]. This narrative review outlines the current state of EAET in treating PPS.

## Theoretical background

EAET has its roots in short-term psychodynamic therapies. However, EAET also draws from several other theoretical frameworks, including affective and pain neuroscience, trauma-focused therapies (e.g., emotional disclosure, trauma rescripting), emotion-focused therapy (e.g., techniques such as the empty chair), and even behavioral therapies (e.g., exposure therapies, assertiveness training). Its goal is to reduce or eliminate fear-driven somatic symptoms by reducing fear of emotionally-laden experience and interpersonal interactions.

### Influence from affective and pain neuroscience

Modern neuroscience provides evidence that the perception of chronic or persistent pain is amplified or generated in the central nervous system and strongly influenced by predictive processing, with input of emotional, cognitive, and social processes [5]. PSS and chronic (primary) pain are viewed as generated by neural processes intimately tied to emotions, particularly the avoidance (e.g., suppression, repression) of adaptive interpersonally-oriented emotions such as anger, sadness/grief, and tender/close feelings, as well as positive self-oriented emotions such as pride and self-compassion. In general, patients with PSS show difficulties in encoding, expressing, and regulating emotions [6]. There is preliminary experimental evidence that implicit (potentially unconscious) negative emotions increase pain unpleasantness [7]. Different models conceptualize the relationship between emotions and somatic symptoms. According to the emotional processing model (EPM), distressing events trigger physiological responses, which typically return to baseline [8]. However, when emotional processing is blocked—due to avoidance, suppression, or a lack of emotional awareness (alexithymia)—emotions remain unresolved and manifest as somatic symptoms or heightened reactivity. Others have proposed a predictive coding model of the development of somatic symptoms, with low interoceptive sensitivity and threat processing strategies due to trait negative affect as relevant factors. Based on the “three process model of implicit and explicit emotion” [9], alexithymia—which is common, for example, in people with fibromyalgia [10]—has been redefined as a phenotype of any combination of several individual mechanisms related to affective response generation, affective response representation, and/or conscious access to the affect [11]. Moreover, there is some evidence that emotional and physical pain share significantly overlapping neuroanatomical pathways [12]. In chronic pain, there is a shift from mainly somatosensory regions to a complex interaction with predominance of affective and cognitive neuroanatomical pathways [13]. EAET takes advantage of these various conceptualizations and the neuroplasticity: by addressing the emotional drivers of pain and fostering emotional processing, the brain’s threat activation system is deactivated (“rewired”), leading to a reduction of the experience of pain and other somatic symptoms [14].

### Roots in psychodynamic theory

EAET has its roots in psychodynamic theory, which emphasizes unresolved developmental trauma, such as neglect, abuse, attachment problems, and other psychosocial conflicts—throughout the life course—as a driving force for PPS [2, 15]. Various somatic symptoms can be initiated earlier in life and often will manifest at a later point in life in response to triggers with psychological links to past experiences.

EAET uses the psychodynamic model of the two triangles, especially the “triangle of conflict” but also the “triangle of person,” to understand the dynamics of emotions, defenses, symptoms, and interpersonal relationships [16]. The “triangle of conflict” describes the interaction between adaptive feelings, defenses, and inhibitory symptoms. People have adaptive, healthy feelings that seek experience and expression: most commonly, anger in response to injustice or victimization, sadness/grief in response to loss, tender/close feelings toward attachment objects, and positive self-oriented feelings such as pride or compassion. Yet, due to maladaptive learning experiences, these emotions are defended against using a range of cognitive, behavioral, and emotional strategies. When experiences activate avoided adaptive feelings, and/or defenses are not effective, distressing emotions or unmet needs may trigger a nervous system response, leading to commonly reported (inhibitory) symptoms such as anxiety, shame, depression, fatigue, numbness, pain, and other somatic symptoms. The “triangle of person” describes interpersonal difficulties and the phenomenon of transference. Thus, the above-mentioned patterns begin with past persons, are maintained with current persons, and are often enacted with the therapist. Therefore, unresolved trauma contributes to problematic relationships marked by an imbalance of assertion and connection, and such “stressful” relationships further activate feelings that are defended against, augmenting the symptoms. Somatic symptoms can also function as defenses and can sometimes be conceptualized as part of the body’s defense mechanism [17]. Thus, EAET involves emotional awareness and experiencing exercises, where individuals are encouraged to identify, experience in the body, label, and give some verbal and bodily expression to adaptive underlying emotions.

## Core treatment processes of EAET

**Fig. 1:**
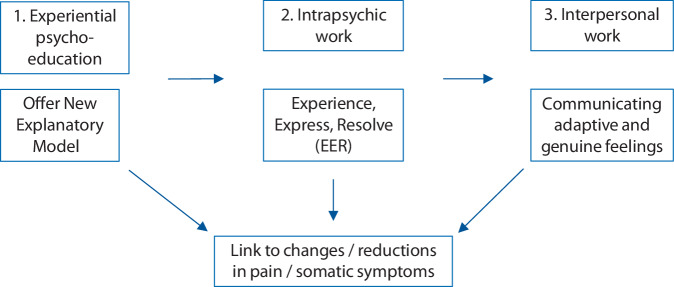
Core treatment principles of emotional awareness and expression therapy (EAET)

EAET is guided by three interdependent core treatment processes. These are reframing symptom explanations to reduce fear, fostering emotional processing, and facilitating corrective interpersonal experiences (Fig. 1).

### Treatment process 1

*Gaining* *a new explanatory model for symptoms to reduce fear and instead experiencing safety will reduce somatic symptoms* *.*

EAET involves helping patients adopt a new explanatory model. Patients with PPS often attribute their somatic symptoms to bodily causes, and their emotional problems to the noxious and disruptive character of these symptoms. As a result, patients routinely avoid their somatic symptoms and emotions, leading to a rather “inhibited life.” Therefore, EAET helps patients see that—although their somatic symptoms are undoubtedly real and experienced bodily—they are driven primarily by the brain’s top-down processes (as described above), rather than somatic causes [18]. Moreover, patients are assisted to see that their emotions—and how they avoid them—are the main drivers of their brain’s symptom activation processes.

By providing psychoeducation and experiential exercises such as imaginal activation of painful behaviors, emotional provocation, and somatic symptom tracking, patients begin to understand that their potentially frightening symptoms can even be helpful—alerting the person to identify and express their feelings and needs. Creating experiences that alter fear-based beliefs and predictions of danger is central to this approach.

#### Treatment process 2

*Intrapsychic work: avoided emotions contribute to physical symptoms; experienced and expressed emotions heal*.

**Fig. 2:**
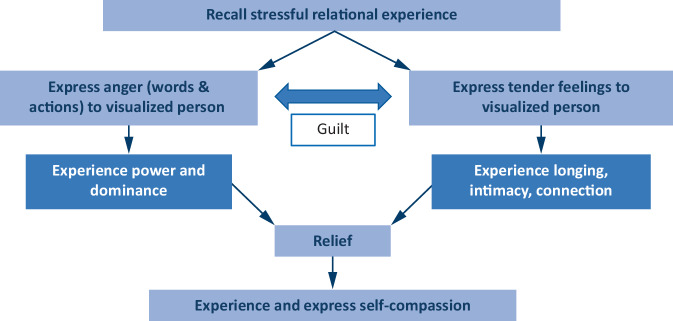
The approach of experience, express, and resolve

EAET aims to facilitate emotional awareness, experiencing, labeling, expression, and eventually, resolution. The “experiencing, expressing, resolving” (EER) technique is central to this process and involves different structured steps (Fig. 2):

- Exploring and expressing anger in conflictual relationships, while dismantling defenses against this emotion.
- Examining guilt related to the anger and grief about the relationship’s state.
- Identifying and expressing longing for closeness and love toward the significant other.

Commonly, the therapist follows the above-mentioned approach. In some cases, it is necessary to start with attachment feelings and then focus on anger, and in other cases, only processing anger, or only grief is to be preferred. Sometimes the entry into the EER process is facilitated by expressing positive self-feelings (e.g., compassion), which may also be used at the end, if there is residual guilt, self-criticism, or sadness.

This stepwise approach in EER aims to help individuals process complex emotional experiences, foster emotional clarity, and develop constructive strategies for addressing relational conflicts, and ultimately reduce PSS. Recent studies on EAET show that greater emotional processing is linked to reduced somatic symptoms [19], even when accounting for depressive symptoms as an alternative mediator [20].

### Treatment process 3

*Interpersonal work: communication of adaptive feelings to significant others may lead to corrective emotional experiences.*

The third process focuses on fostering corrective emotional experiences in relationships, a concept introduced by Franz Alexander [21]. EER (second principle) leads to increased awareness of one’s interpersonal needs and motivates the person to modify their relationships. This involves expressing both needs of assertiveness and connection. Patients have to find some healthy point, which could range from firmer boundaries (to the point of ending relationships) to investing in increased closeness and intimacy. Doing so helps to unlearn fears of these emotions, thereby further reducing somatic symptoms, as well as potentially changing core sources of stress or conflict. This principle involves both intrapsychic work (resolving inner conflicts) and interpersonal work (engaging with significant others).

When patients desire to try to heal a relationship, they are encouraged to confront and express avoided emotions—such as anger and love—toward important figures in their lives, but do so in a thoughtful, balanced manner and in consideration of the needs and feelings of the other person. In-session EER exercises are often emotionally intense, which not only reduces fear of emotions but can foster the patients’ awareness of their feelings and needs, and consequently increase their motivation to communicate them in relationships. Homework exercises, like writing unsent letters, can facilitate this process, too. Patients can also practice this by interacting with their therapist in new ways, such as asserting their needs and expressing feelings of gratitude [22], where, it is hoped, they will create new relational patterns and further reduce their PPS.

## Evidence for efficacy from controlled trials

EAET has been developed and evaluated in various formats, including individual therapy, group therapy, and internet-based interventions. Moreover, the number of sessions has varied from a single session to 10. Since 2017, multiple randomized controlled trials (RCTs) have assessed the efficacy of EAET. Here, we briefly review single-session trials, group interventions, and guided internet administrations. (Another trial demonstrated the efficacy of EAET delivered individually in three sessions to people with irritable bowel syndrome [22]).

### EAET as an individual one-session intervention

Using a protocol called the life stress interview (LSI), EAET has been tested as a single-session, 90 min intervention. In a RCT involving 75 patients with medically unexplained symptoms in primary care, adding the LSI to usual care significantly reduced pain intensity (d = 0.62) and pain interference (d = 0.71) compared to a waitlist/usual care condition [23]. In another RCT of 62 women with chronic urogenital pain, the LSI also led to greater pain reduction (partial η^2^ = 0.07) than waitlist/usual care [24]. In a third study with patients with chronic musculoskeletal pain and elevated childhood adversities, the EAET psychodynamic interview reduced pain inference (partial η^2^ = 0.05) and anxiety (partial η^2^ = 0.04), though the significant decrease in pain intensity after the interview was not greater than the decrease in pain in the waitlist controls [25]. Overall, these findings support the potential clinical value of an EAET interview in reducing pain and improving well-being in patients with PPS, despite being a single-session intervention. However, all studies employed short follow-up periods (6 weeks), and it remains unclear whether these effects are sustained over a longer period or how this EAET interview compare to active controls.

### EAET administrated as group therapy

Group-based EAET usually involves 8–10 weekly sessions, each lasting 90 min, with 5–7 participants per group. This format utilizes not only the greater efficiency of group therapy but possibly the power of interpersonal disclosure, modeling, and support. In a landmark RCT, 230 patients with fibromyalgia (FM) where randomized to EAET, cognitive–behavioral therapy (CBT), or an active control condition: FM education [26]. EAET led to significantly lower FM symptoms (between-condition d = 0.35) and widespread pain (d = 0.37) than CBT. EAET had significantly better outcomes than FM education (between-condition d’s ranging from 0.29 to 0.45). In a RCT of 53 older military veterans with musculoskeletal pain [27], group EAET produced significantly lower pain severity than group CBT at posttreatment and 3‑month follow-up; differences were large (partial η^2^ = 0.129 and 0.157, respectively). This result was replicated in a follow-up trial with a larger sample of 126 older veterans with musculoskeletal pain [28]. In sum, these three studies show some superiority of EAET over CBT. Moreover, across these trials, an average of 30% of patients experienced a reduction in their pain by 50% or more after the group EAET, but only an average of 5.5% in the CBT group. A recently published trial, published in Persian but summarized in English, reported that group EAET was superior to both no treatment and group acceptance and commitment therapy in reducing both pain and anger among 75 women with breast cancer [29]. The evidence from these RCTs highlights that EAET group therapy is an effective approach for pain reduction in fibromyalgia and chronic musculoskeletal pain.

### EAET as internet-administered guided self-help

To increase accessibility, EAET has been adapted into an internet-based, guided self-help format (I-EAET). This version includes 10 modules that combine psychoeducation, experiential exercises, and therapist support via email. In a controlled trial, 74 participants were randomized to I‑EAET or a wait-list [20]. Although the treatment effects of I‑EAET were smaller compared to an earlier uncontrolled trial [30], I‑EAET showed promise as an intervention for PPS, with a near medium between-condition effect size for somatic symptom reduction. Approximately 20% of patients experienced significant clinical improvement (at least 50% symptom reduction), indicating its effectiveness for a subset of individuals. The effects were largely maintained at the 12 month follow-up [31]. In sum, I‑EAET shows promising results, with a subset of patients achieving significant symptom reduction.

## Case example

Anna, a 40-year-old woman with chronic back pain, underwent extensive medical evaluations without finding a somatic cause. Alongside her pain, she experienced anxiety and depression, particularly following her father’s death three years prior, which disrupted her life and relationships.

During her first EAET session, Anna underwent a detailed exploration of her health and psychosocial history, particularly exploring potential links between unresolved emotional experiences and her somatic symptoms. Anna reported that recent spinal imaging found disc anomalies, but the therapist explained these as typical for most people her age and not the main pain driver. This psychoeducational reframing helped reduce her fear of pain and opened the door to exploring emotional processing. Notably, it was observed that Anna had experienced stomach pain since childhood while growing up with an alcoholic father.

### Core treatment elements

1. New symptom model
  Anna learned by experiential exercises how predictive coding and sensitized neural pathways amplified her pain based on emotional triggers. This reduced her catastrophic thinking and encouraged emotional engagement.
2. Emotional processing (EER technique)
  During imaginal exercises, Anna was helped to express strong anger toward her father for neglecting her as a child. This expression of suppressed feelings led to the experience and processing of guilt, and subsequently grief and longing. She worked through these conflicted feelings, and then practiced self-compassion, which helped transform her self-critical pattern.
3. Corrective interpersonal experiences
  Between sessions, Anna practiced healthy communication, such as expressing boundaries to her partner and fostering intimacy with her mother. These actions enhanced her sense of agency and connection and further helped her reduce her fear of these emotions and their expression.
  Emotional breakthroughs, such as expressing anger toward her father with her words and fists and crying while mourning the loss of a safe childhood environment, allowed for corrective experiences, where the therapist validated her feelings in ways her father had not been able to.

### Outcome

By the end of treatment, Anna’s pain decreased substantially, her anxiety and depression improved, and she developed emotional awareness and self-compassion. She described her journey as transformative, highlighting EAET’s ability to address emotional underpinnings of PPS and promote meaningful healing.

## Potential and gaps

This narrative review presents the theoretical rationale, core treatment principles, and empirical efficacy of EAET, an integrative therapeutic approach grounded in psychodynamic theory, with influences from affective neuroscience and emotion-focused therapy. EAET’s theoretical foundation emphasizes unresolved emotional trauma and psychosocial conflict as a contributing factor to persistent physical symptoms (PPS). Through this review, the importance of emotional processing—reversing emotional avoidance by activating, experiencing, expressing, and resolving trauma and conflict—in alleviating PSS has been highlighted.

When integrating the findings from RCTs of EAET in different treatment formats, a rather compelling case emerges for the use of EAET to treat patients with PSS—such as chronic primary pain—that are driven primarily by the central nervous system. Importantly, three trials—of all group EAET—have demonstrated its superiority to CBT in reducing pain, and such demonstrations of superiority over bona fide alternatives are rare. We think that EAET’s efficacy, might be due to two core principles present in EAET: First, EAET adopts a model that views PSS not as a chronic or lifelong condition due to primarily irreversible somatic etiology, which one has to cope with, but rather as a largely brain-based in the sense of predictive processing that can be changed or reversed. Second, EAET directly addresses the emotional and interpersonal drivers of PSS that result from trauma or psychosocial conflict by supporting the processing and resolution of such. Additionally, EAET’s versatility is a notable strength, as it has been successfully developed and delivered in various formats, including one-session interventions, individual therapy, and internet-administered programs, broadening its accessibility and adaptability.

However, several gaps in the research remain [32]. One pressing need is to assess the long-term efficacy of EAET, as studies on its sustained benefits across diverse patient populations are limited. Also, although EAET has shown significant somatic symptom reductions compared to other active treatments, these effects are seen in only a subset of patients. This highlights the need for further research into predictors and moderators of treatment outcomes to better understand which patients are most likely to benefit from EAET. In addition, EAET is—compared to most psychological therapies for PSS—emotionally challenging for patients and often for therapists. It will be valuable to better understand when anxiety regulation is needed and to determine the intensity of emotional work that each patient can adaptively make use of before exceeding their regulation threshold.

There also is a need for studies that test the mechanisms of action most important to therapeutic outcomes of EAET. Like most interventions, EAET contains many specific possible mechanisms, including a shift in patient’s understanding of pain, emotional disclosure of private or secret experiences, emotional experiencing and expression, insight into patterns, changing interpersonal patterns, and often a deep or intensive alliance with therapists that need to be negotiated. Finally, studies would benefit from using not only self-report measures of symptoms and functioning, but also including biological and brain changes and behavior changes, including changes in healthcare utilization, medication use, and disability status. Despite these areas of needed research to improve tailored treatment, EAET shows considerable promise for treating patients with PSS, especially those with chronic primary pain.

## Practical conclusions

- Emotional awareness and expression therapy (EAET) is a feasible and effective treatment for patients with persistent physical symptoms, particularly chronic primary pain, and appears to be more effective than cognitive–behavioral therapy (CBT).
- EAET can be delivered in individual, group, and internet formats, offering flexibility for different patient needs.
- Emotional processing—identifying, experiencing, and expressing adaptive emotions towards oneself and significant others—appears to be a key mechanism to reducing persistent physical symptoms (PPS).

## Declarations

This review did not involve any patient data; therefore, no ethical approval was required. The case of Anna is a composite presentation of many patients created to illustrate a typical treatment scenario of EAET.
